# Heart Rate Variability in Healthy Subjects During Monitored, Short-Term Stress Followed by 24-hour Cardiac Monitoring

**DOI:** 10.3389/fphys.2022.897284

**Published:** 2022-06-13

**Authors:** Zifan Gu, Vanessa C. Zarubin, Katherine R. Mickley Steinmetz, Carolyn Martsberger

**Affiliations:** ^1^ Department of Physics, Wofford College, Spartanburg, SC, United States; ^2^ Department of Biomedical Informatics, Harvard Medical School, Boston, MA, United States; ^3^ Psychology Department, Northwestern University, Evanston, IL, United States; ^4^ Department of Psychology, Wofford College, Spartanburg, SC, United States

**Keywords:** heart rate variability, detrended fluctuation analysis, acute stress, time series, parasympathetic, 24 h holter

## Abstract

Heart Rate Variability (HRV) can be a useful metric to capture meaningful information about heart function. One of the non-linear indices used to analyze HRV, Detrended Fluctuation Analysis (DFA), finds short and long-term correlations in RR intervals to capture quantitative information about variability. This study focuses on the impact of visual and mental stimulation on HRV as expressed *via* DFA within healthy adults. Visual stimulation can activate the automatic nervous system to directly impact physiological behavior such as heart rate. In this investigation of HRV, 70 participants (21 males) viewed images on a screen followed by a math and recall task. Each viewing segment lasted 2 min and 18 s. The math and memory recall task segment lasted 4 min total. This process was repeated 9 times during which the participants’ electrocardiogram was recorded. 37 participants (12 males) opted in for an additional 24-h Holter recording after the viewing and task segments of the study were complete. Participants were randomly assigned to either a pure (organized image presentation) or mixed (random image presentation) image regime for the viewing portion of the study to investigate the impact of the external environment on HRV. DFA *α*1 was extracted from the RR intervals. Our findings suggest that DFA *α*1 can differentiate between the viewing [DFA *α*1 range from 0.96 (SD = 0.25) to 1.08 (SD = 0.22)] and the task segments [DFA *α*1 range from 1.17 (SD = 0.21) to 1.26 (SD = 0.25)], *p* < 0.0006 for all comparisons. However, DFA *α*1 was not able to distinguish between the two image regimes. During the 24-hour follow up, participants had an average DFA *α*1 = 1.09 (SD = 0.14). In conclusion, our findings suggest a graded response in DFA during short term stimulation and a responsiveness in participants to adjust physiologically to their external environment expressed through the DFA exponent.

## 1 Introduction

The recorded electrical signal from the heart known as an electrocardiogram (ECG) has been reported for over a century (Barold, 2003) and can be an informative, minimally invasive measure of the heart’s function (Lach et al., 2021). An ECG has distinctive features such as the R peaks in the QRS complex that are readily identifiable and analyzable. The successive R peaks known as RR intervals provide the basis of heart rate variability (HRV) analysis (Kuusela, 2013). These changes in the successive heartbeats are an integral component of physiological research and are becoming more standardized in the field (Laborde et al., 2017; Berntson et al., 1997; Malik, 1996) to provide diagnostic information in certain medical conditions (Yeh et al., 2009; Gronwald et al., 2019) and insights into the parasympathetic nervous system (Malik, 1996). Past studies have shown that shifts in HRV can be induced by a variety of different types of affective stimuli such as watching positive and negative videos (Barquero-Pérez et al., 2020; Ghiasi et al., 2020), the Cold Pressor Stress Test (Ghiasi et al., 2018; Ghiasi et al., 2020), and imagining scenarios which evoke an emotional response (McCraty et al., 1995). Using these different affective induction techniques, researchers have proposed various models to measure changes in the autonomic nervous system in reaction to an affective stimulus. An increase in HRV suggests an increase in executive function and mobility efficiency, whereas a decrease in HRV suggests a higher risk for disease and mortality (Shaffer et al., 2020). Methods used to analyze HRV include time domain analyses such as the root mean square of successive RR interval differences (RMSSD), the percentage of successive RR intervals differing by more than 50 ms (pNN50), and the standard deviation of the normal-to-normal intervals (SDNN) and frequency domain analyses such as ultra-low-frequency (ULF), very low frequency (VLF), low-frequency (LF), and high-frequency (HF) power (Shaffer and Ginsberg, 2017). Non-linear methods include SD1 (Raetz et al., 1991; Tulppo et al., 1996), Approximate Entropy (ApEn) (Pincus, 1991), and Detrended Fluctuation Analysis (DFA) (Peng et al., 1995). In this study, we investigated how visual stimulation and mental stress tasks impact HRV using DFA. First used in DNA sequences (Peng et al., 1993), DFA is one of the most widely used non-linear methods to analyze HRV because it can both decrease the impact on noise while identifying meaningful, local trends (Cui et al., 2020).

Historically, HRV analysis can be derived from a variety of environments including 24-hour monitoring and short-term recordings under various external conditions (Shaffer and Ginsberg, 2017). However, the long-term data collection process is inconvenient, expensive, can delay a diagnosis and in some cases may not be feasible (Heitmann et al., 2011). While low-intensity physical tests can serve as stimuli for research in cardiac patients, activities like running on a treadmill may not be practical in some patients with underlying cardiac conditions. To address these limitations, short-interval and ultrashort-interval HRV methods provide informative and practical assessment options (Shaffer et al., 2016; Shaffer et al., 2020). In addition to capturing shorter time series of data, research has shown that participants performing more passive tasks, such as watching movies, can be useful (Iyengar et al., 1996). By limiting the physical motion of our participants and stimulating *via* a programmable computer screen, we designed a repeatable and consistent external environment while attempting to minimize differences due to individual movement. This provides a two-fold benefit for HRV analysis by shortening the time of recordings and providing an ultrasafe, accessible environment for patients to engage in.

DFA as a non-linear measurement of HRV can provide insights into relatively short time series as recent studies have suggested (Hautala et al., 2003; Yeh et al., 2009; Heitmann et al., 2011; Gronwald et al., 2019). The detrended characteristics of the time series derived from this method allows ECG recordings to be succinct while maintaining the quality of the analysis (Hautala et al., 2003). Moreover, this makes DFA independent of heart rate to account for the different backgrounds of a study population including fitness level, nutrition, and a balanced lifestyle (Gronwald et al., 2019). A previous study showed a consistent, alternating pattern of DFA *α*1 values in healthy participants engaged in high intensity exercise training followed by active recovery. This alternating pattern in DFA *α*1 values persisted over the course of the study while the engagement in high intensity training and active recovery was repeated (Gronwald et al., 2019). Similarly, if healthy participants can also achieve consistent, alternating changes in DFA *α*1 values between mental stress tasks and viewing images, this setup may provide complementary insight into HRV alongside traditional exercise tests. Specifically, the ability of a healthy participant to switch DFA *α*1 values consistently and repeatedly in response to a series of alternating, external triggers may indicate good health. Moreover, because previous studies showed that DFA *α*1 values tend to be much less than 1 in high-risk cardiac patients (Peng et al., 1995; Huikuri et al., 2009), the absence of a graded response in DFA *α*1 values to an external environment may offer prognostic clinical value that is correlated with the loss of responsiveness in fractal organization. To study this, we explored the impact of external factors on HRV (Krstacic et al., 2007). We placed healthy participants in short-term stress situations and monitored 24-hour cardiac behavior in a subset of the study population. Overall, we investigated how the external environment such as viewing images in different sequences and performing mental tasks impacted HRV.

## 2 Materials and Method

### 2.1 Participants

73 undergraduate students between the ages of 18 and 22 from Wofford College were enrolled in this study for compensation of either course credit or merchandise gift cards. Participants with pulmonary embolism (*n* = 1) and tachycardia (*n* = 2) were excluded. Each person was randomly assigned to either a pure image regime (*n* = 34, 12 males) or a mixed image regime (*n* = 36, 9 males) during Phase I of the study. The description of a pure image regime and a mixed image regime, as well the phases, are presented in the next sections. Out of the 70 participants (21 males), 37 (12 males) participated in the optional Phase II of the study. Participants did not report a history of drug or alcohol abuse, were not under general anesthesia in the 2 weeks prior to testing and did not experience a traumatic physical event within 30 days of the testing. Past medical history was collected from questionnaires for each participant prior to the start of the Phases. Although recent physical activities prior to the start of Phase I were not recorded, participants needed between 1 and 2 h to be set up for the experiment. During most of this time, participants were sitting quietly in the testing room. This provided a uniform, baseline condition across participants before Phase I began (Javorka et al., 2002). Our participants were a subset from the same population of a previous study focusing on brain activity (Aguillard et al., 2020). All participants provided written informed consent, and procedures were approved by the Wofford College Institutional Review Board.

### 2.2 Study Materials

Participants in this study were randomly assigned to two image regimes: the pure image regime and the mixed image regime. In the pure regime, participants viewed images from the same image type for a total of nine segments presented in the following order (Table 1). In the mixed regime, participants viewed a mixture of images from all three image types for a total of nine segments. The images in these segments were categorized as one of the following types: negative, neutral, and categorical. Each type of image had different ratings in arousal, valence, and relatedness. Arousal and valence ratings were determined in pilot studies where participants ranked each image on a 9-point Likert scale from “calm/soothing” to “exciting/agitating” (arousal ratings); and from “very unpleasing” to “very pleasing” (valence ratings). Relatedness ratings were ranked on a 7-point Likert scale from “low association” to “high association” (Zarubin et al., 2020). Negative images were rated as significantly more unpleasant and higher in arousal than the neutral images (e.g., a hospitalized man, a snake, or a boy running from a sniper). Neutral images were rated in the middle of the valence scale from pleasant or unpleasant, low in arousal, and low in relatedness (e.g., a man by a car, a cup on a table, or a boy playing chess). Categorical images were rated in the middle of the valence scale from pleasant or unpleasant, low in arousal, and high in relatedness (e.g., an entrance way, a kitchen scene, a man cooking in a kitchen).

**TABLE 1:** Types of images participants saw in the pure regime during each segment.

Seg. 1	Seg. 2	Seg. 3	Seg. 4	Seg. 5	Seg. 6	Seg. 7	Seg. 8	Seg. 9
Negative	Categorical	Neutral	Categorical	Neutral	Negative	Neutral	Negative	Categorical

To create the image regime viewing sequence of pictures, 66 negative images were selected from 150 negative images; 66 neutral images were selected from 100 neutral images; and 66 categorical images were selected from 150 categorical images from the International Affective Picture System (IAPS) (Lang et al., 1999), the Geneva Affective Picture Database (Dan-Glauser and Scherer, 2011), the Emotional Picture Set (Wessa et al., 2010), the image pool of Talmi et al. (2007), and Google Images.

### 2.3 Procedure

**FIGURE 1:**
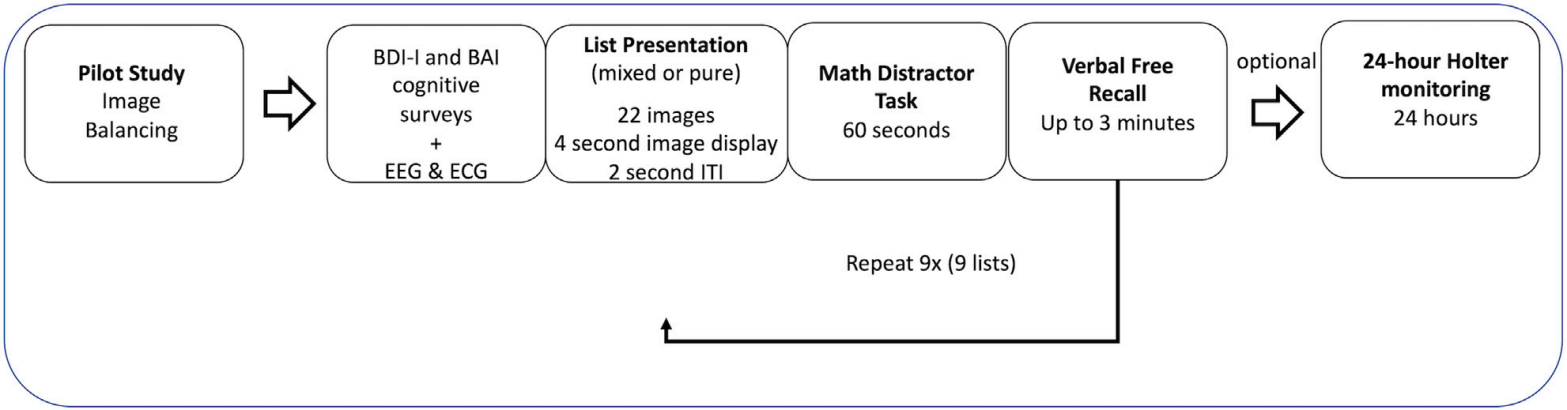
Experimental timeline.

The experiment consisted of two phases: Phase I and Phase II. Phase I was a controlled environment where participants viewed images and performed tasks by instruction. Phase II was a semi-controlled environment where participants followed guidelines to resume normal activities. An ECG was attached to participants throughout both phases (model specifications below). Prior to start of Phase I, participants completed the depression and anxiety questionnaires (BDI-I and BAI) and a practice test. The experimental time can be found in Figure 1.

#### 2.3.1 Phase I

Phase I of the study was composed of 9 segments with 22 images in each segment. Every segment could have images from within the same group (the pure image regime) or a combination of all groups (the mixed image regime) depending on the regime the participant was assigned to. Each segment lasted 2 min and 18 s. Each image was active for 2 s followed by a 4 s fixation cross to reduce the emotional stress that occurred during the previous image (Talmi and McGarry, 2012). Participants were asked to view the images in an upright sitting posture. This section of Phase I is referred to as “viewing” throughout this manuscript. At the conclusion of each segment (i.e., after the 22 images were viewed), each participant was asked to perform a 1-minute arithmetic task followed by a memory recall task for up to 3 min. Participants reported verbally to research assistants on what they saw during the previous viewing segment. This results in a variable RR interval length which is addressed later in this paper. This section of Phase I is referred to as “task” throughout this manuscript. The controlled environment of Phase I (viewing and tasks) was designed to eliminate physiological differences between participants during their resting state (Iyengar et al., 1996). The duration of Phase I was approximately 1 h. A 3-lead ECG was acquired *via* 3 flat-type electrodes (DA-AT-EXTOF1) attached to the EXG channels of the Cortech Active Two 32 Channel EEG system (Manufacturer: Cortech Solutions; Model Specifications: Model Number DA-AT_HCL32) from the Behavioral Brain Sciences Center, Birmingham, United Kingdom. Phase I data was sampled at 1.024 kHz and Lead III was primarily used for analysis.

#### 2.3.2 Phase II

Phase II of the study was optional and started approximately 1 h after the conclusion of Phase I. During this phase, participants wore an ECG recording device (BIOPAC Systems, Goleta, CA, Model Number BN-LOG, 3 Lead ECG) while carrying out normal daily activities for 24 h. Phase II data was sampled at 1 kHz, and Lead I was primarily used for analysis.

### 2.4 Data Processing

The 24-hour ECG data obtained from Phase II was first reviewed in the BIOPAC systems accompanying software (AcqKnowledge) and then exported to MATLAB for additional cleaning. In some cases, 24-hour data was directly exported to MATLAB when access to the AcqKnowledge software was unavailable. Under either condition, MATLAB was used to plot raw ECG voltage data. Research assistants were trained to identify ECG signals *via* visual inspection (i.e., every signal was checked for its integrity and signal quality). If a signal had to be removed because the QRS complex could not be identified, the signal was removed in such a way that the cut began and ended with the same component of the QRS complex (i.e., from the peak of one QRS wave to the peak of another or from the beginning of one P wave to the beginning of another), similar to other studies (Hautala et al., 2003; Yeh et al., 2009; Krolak, et al., 2020; Mizobuchi et al., 2021). For the ECGs collected in Phase I, data was also cleaned in MATLAB in the same identical manner as Phase II. After all signals were cleaned, custom written MATLAB code was used to identify RR peaks. P peaks and T peaks that were mistakenly identified as R peaks by MATLAB were also corrected by research assistants by removing those entries and marking the correct R peaks manually. Missing peaks were not interpolated. Of the 2 min and 18 s (viewing) and up to 4 min (tasks) for each segment, the first 2 minutes were used for analysis. Note that since participants were required to take an arithmetic test during the first minute of tasks, the 2 minutes of tasks in the analysis contained a 1-minute arithmetic test and a 1-minute memory recall. Participants missing more than 5% of the recording were excluded from the segment of the analysis. The number of participants excluded in each segment is presented in Table 2.

**TABLE 2:** Number of participants excluded out of 70 for DFA *α*1 analysis.

	Seg. 1	Seg. 2	Seg. 3	Seg. 4	Seg. 5	Seg. 6	Seg. 7	Seg. 8	Seg. 9
Viewing	7	2	14	2	10	3	2	5	2
Task	15	11	10	8	11	8	12	9	11

Of the collected 24.0 h of Phase II data, 15 participants were excluded for having less than 22.8 h of recordings (i.e., missing more than 5% of their recordings). The first 20 h of RR intervals of the remaining 22 participants were used for analysis. DFA *α*1 index of HRV were then computed on the qualifying data. DFA can quantify the fluctuations of interbeat intervals (Shaffer et al., 2016) by looking at regions (i.e., different window sizes) successively within short intervals and thus is suitable for analysis of this data. Given the short time interval of each segment, only DFA *α*1 is reported.

### 2.5 The Detrended Fluctuation Analysis

In each time series, DFA *α*1 is calculated by dividing segments into window sizes (*n*) of 4–16 (Peng et al., 1995) and subtracting a linear fit from within that window to detrend the data. The fluctuation, *F*(*n*), is the root mean square of the detrended time series. It can be calculated as:

$$F\left(n\right)=\;\sqrt{\frac{1}{N}\sum_{k=1}^{N}[y\left(k\right)-y_{n}{(k)]}^{2}}$$

Where N is the length of the time series, $y_{n}\left(k\right)$ is the local trend within each window, and y(k) is the value of the integrated time series (Peng et al., 1995). The scaling component, DFA *α*1, is the ratio of *F*(*n*) over window sizes (*n*) on a log-log plot. An *α* of 0.5 implies that the fluctuation is independent of any factors, an uncorrelated random walk. An *α* of 1.5 is Brownian noise (Peng et al., 1995). An *α* of 1 implies a perfect log-log correlation and is generally found in healthy subjects (Kuusela, 2013). This healthy behavior corresponds to the general theory of chaos theory: There are deterministic mechanisms that appear random but offer some predictability (Goldberger, 1996). As *α* increases from 1, it suggests a decrease in fluctuation and thus deviates from the randomness presented in chaos theory (Goldberger, 1996). Furthermore, the short-term correlations extracted through DFA may reflect a response of the baroreceptor reflex, while the long-term correlations reflect other regulatory mechanisms that moderate fluctuations (Shaffer and Ginsberg, 2017).

In Phase I data, DFA *α*1 of each participant was computed by finding the ratio of log *F*(*n*) vs. log *n* for 4 ≤ *n* ≤ 16; in Phase II data, segments were divided into 2-minute periods (the length of each image segment from Phase I) as a single 24-h time series analysis can overlook the different activities during the day and violate stationarity (Malik, 1996; Laborde et al., 2017). DFA for a participant was then computed by taking the arithmetic mean of the individual subset DFA values (Mizobuchi, 2021). This method was implemented to address the concern (Fei et al., 1996; Shaffer et al., 2020) of low correlation for comparing DFA between short term (a few hours) and long term (24 h) data. During the 24 h, there could be fluctuations in cardiac behavior that arise due to differences between sleeping and eating, for example. However, because participants were asked to resume normal activities during Phase II, we approximate that any 2-minute bin of data captures a roughly uniform period of activity. One of our goals in our analysis of Phase II was to understand this 2-minute bin of ECG data when participants were not stimulated by the viewing images or performing tasks as they were in Phase I.

The computation of the DFA value was done using custom written MATLAB code (Magris, 2022). The algorithm was checked manually to ensure that the average, summation, and variances of the local trend were calculated correctly. The algorithm was also verified on pre-analyzed data intervals (Goldberger et al., 2000) to match the expected output.

### 2.6 Data and Statistical Analysis

Of the 2 minutes analyzed in tasks, the analysis contained a 1-minute arithmetic test and a 1-minute memory recall. While both tasks served as a distractor from previous images, the nature of the tasks may bring insight about the extent of DFA *α*1’s predictive power, because past work showed that adding social evaluation increases physiological symptoms of stress in participants (Schwabe et al., 2008). Thus, RR intervals were analyzed with increasing temporal resolution of a segment to take this into account.

The first resolution compared each activity to its subsequent activity at the 2-minute scale. For example, the 1^st^ segment of the viewing was compared to the 1^st^ of the task segment. The 1^st^ segment of task was then compared with 2^nd^ segment of viewing, etc. for a total of 17 comparisons. In addition, each viewing and task segment was compared to their subsequent equivalent segments. That is, the 1^st^ segment of viewing was compared to the 2^nd^ segment of viewing; the 1^st^ segment of task with 2^nd^ segment of task, and so on. The second resolution compared each minute of activity to its subsequent minute/activity. For example, the 1^st^ minute of the 1^st^ segment of viewing was compared with the 2^nd^ minute of the 1^st^ segment of viewing, the 2^nd^ minute of the 1^st^ segment of viewing was compared with 1^st^ minute of the 1^st^ segment of task, and so on, for a total of 35 pairs of comparison.

DFA in Phase II data was calculated using a 2-minute scale. DFA *α*1 was calculated for each 2-minute epoch and the 24-hour DFA *α*1 for each participant was the average of all epochs. Paired sample t-test analysis was done using SPSS (IBM SPSS Statistics for Macintosh, Version 26.0).

## 3 Results

**FIGURE 2:**
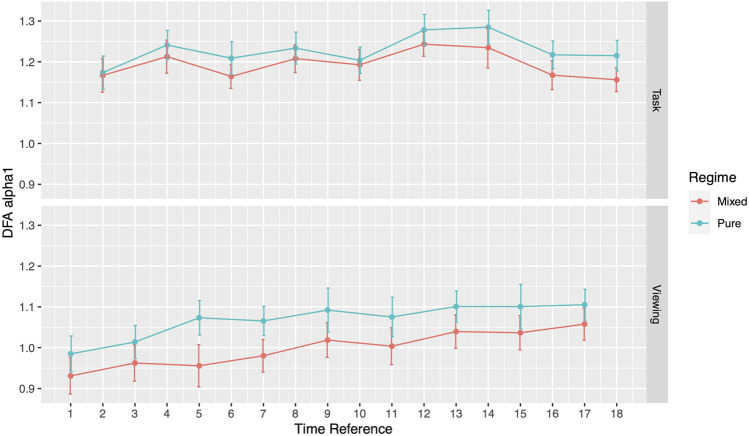
DFA *α*1 for the two image regimes during each activity.

During the viewing section of Phase I, participants assigned to the pure image regime presented no differences in DFA *α*1 compared to the mixed image regime participants. DFA *α*1 for pure regime participants ranged from 0.99 (SD = 0.25) to 1.11 (SD = 0.22), while the range for mixed regime participants ranged from 0.93 (SD = 0.24) to 1.06 (SD = 0.23) (Figure 2; Table 3). Similar results emerged during the tasks section. Specifically, DFA *α*1 for pure regime participants ranged from 1.17 (SD = 0.21) to 1.28 (SD = 0.22), while the range for mixed regime participants was from 1.16 (SD = 0.17) to 1.24 (SD = 0.17) (Figure 2; Table 3). The significance level was corrected for multiple comparisons (Bonferroni correction, *p*-value threshold <0.0056). We also did not find significant differences in the three image types (neutral, negative, or categorical) within the pure regime participants (Supplementary Table S1). Thus, the following results are presented for all participants in both regimes. We will first present our results during Phase I comparing the ECG analysis from the viewing sections to the tasked sections of testing and then present our findings in Phase II.

**TABLE 3:** DFA *α*1 comparison between Pure Regime and Mixed Regime participants in both viewing and task. All numbers except *p*-values are presented as mean (SD).

	Seg. 1	Seg. 2	Seg. 3	Seg. 4	Seg. 5	Seg. 6	Seg. 7	Seg. 8	Seg. 9
PURE view	0.99 (0.25)	1.01 (0.23)	1.07 (0.21)	1.07 (0.20)	1.09 (0.27)	1.08 (0.28)	1.10 (0.21)	1.10 (0.30)	1.11 (0.22)
MIX view	0.93 (0.24)	0.96 (0.26)	0.96 (0.29)	0.98 (0.24)	1.02 (0.25)	1.00 (0.26)	1.04 (0.25)	1.04 (0.25)	1.06 (0.23)
*p*	0.389	0.395	0.085	0.116	0.291	0.283	0.275	0.355	0.387
PURE task	1.17 (0.21)	1.24 (0.18)	1.21 (0.21)	1.23 (0.21)	1.20 (0.17)	1.28 (0.21)	1.28 (0.22)	1.22 (0.19)	1.22 (0.19)
MIX task	1.17 (0.22)	1.21 (0.23)	1.16 (0.17)	1.21 (0.19)	1.19 (0.22)	1.24 (0.17)	1.23 (0.27)	1.17 (0.19)	1.16 (0.16)
*p*	0.908	0.600	0.375	0.622	0.826	0.468	0.441	0.315	0.217

### 3.1 Phase I: Paired Analysis within Subjects Between Viewing and Task Data

**FIGURE 3:**
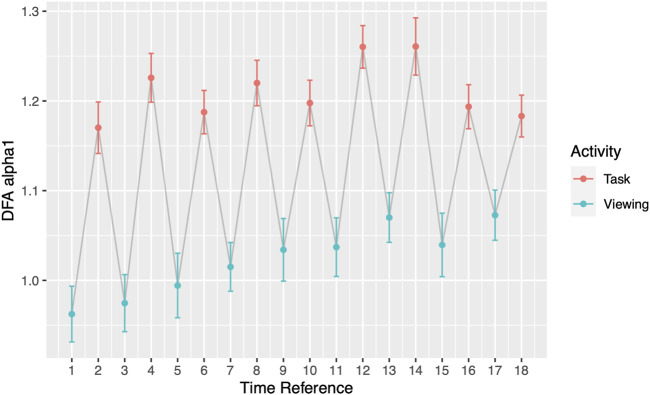
DFA *α*1 by segments. Odd Time References represent Viewing; even Time References represent Task. Grey line connects activities chronologically.

Throughout the study, participants DFA values alternated in sync with the alternating pattern of the viewing and task segments. During Phase I, the comparisons between each activity to its subsequent activity (2-minute resolution) presented significant differences in all 17 pairs (Figure 3; Table 4). DFA *α*1 ranged from 0.96 (SD = 0.25) to 1.08 (SD = 0.22) during viewing and from 1.17 (SD = 0.21) to 1.26 (SD = 0.25) during tasks. On the contrary, within a given segment type (i.e., comparing all viewing segments or all tasks segments), none of the same segments type showed significant differences (Supplementary Table S2). The significance level was corrected for multiple comparisons (Bonferroni correction, *p*-value threshold <0.0029).

**TABLE 4:** DFA *α*1 2-minute comparison during Phase I. *p*-values are reported in comparison with previous activity. All numbers except *p*-values are presented as mean (SD).

	DFA α1	*p*
Viewing 1	0.96 (0.25)	—
Task 1	1.17 (0.21)	<0.0001
Viewing 2	0.99 (0.25)	<0.0001
Task 2	1.23 (0.21)	<0.0001
Viewing 3	1.01 (0.26)	<0.0001
Task 3	1.19 (0.19)	<0.0001
Viewing 4	1.02 (0.22)	<0.0001
Task 4	1.22 (0.20)	<0.0001
Viewing 5	1.05 (0.26)	<0.0001
Task 5	1.20 (0.20)	<0.0001
Viewing 6	1.04 (0.27)	<0.0001
Task 6	1.26 (0.19)	<0.0001
Viewing 7	1.07 (0.23)	<0.0001
Task 7	1.26 (0.25)	<0.0001
Viewing 8	1.07 (0.27)	<0.0001
Task 8	1.19 (0.19)	0.0006
Viewing 9	1.08 (0.22)	<0.0001
Task 9	1.18 (0.18)	<0.0001

**FIGURE 4:**
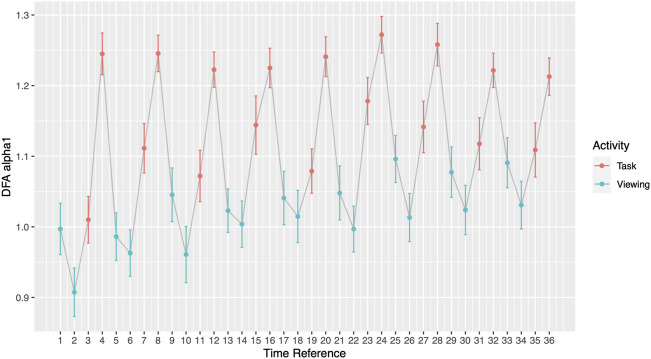
DFA *α*1 by segments at one-minute scale. Grey line connects activities chronologically.

The comparisons between each minute of the activity compared to its subsequent minute (1-minute resolution) presented significant differences. 15 out of 35 minute-to-minute consecutive transitions showed a significant change in DFA *α*1 and 10 of these transitions were significant when they occurred between the viewing segment and a task segment (Figure 4; Supplementary Table S3). 5 out of 9 comparisons between the 2^nd^ minute of task to 1^st^ minute of task presented significant HRV differences between the arithmetic test and memory recalls (Table 5). Out of those 5 significant comparisons, 4 pairs came from the first half of Phase I. 0 out of 9 comparisons presented differences between the 1^st^ minute of viewing and the 2^nd^ minute of viewing (Supplementary Table S4). The significance level was corrected for multiple comparisons (Bonferroni correction, *p*-value threshold <0.0014).

**TABLE 5:** DFA *α*1 one-minute comparison within tasks. All numbers except *p*-values are presented as mean (SD).

	DFA α1	*p*
Task1 min1	1.01 (0.24)	—
Task1 min2	1.25 (0.22)	<0.0014
Task2 min1	1.11 (0.27)	—
Task2 min2	1.25 (0.20)	<0.0014
Task3 min1	1.07 (0.28)	—
Task3 min2	1.22 (0.20)	<0.0014
Task4 min1	1.15 (0.32)	—
Task4 min2	1.22 (0.22)	0.0846
Task5 min1	1.08 (0.24)	—
Task5 min2	1.24 (0.22)	<0.0014
Task6 min1	1.18 (0.26)	—
Task6 min2	1.27 (0.20)	0.0036
Task7 min1	1.14 (0.28)	—
Task7 min2	1.26 (0.23)	<0.0014
Task8 min1	1.12 (0.29)	—
Task8 min2	1.22 (0.19)	0.0018
Task9 min1	1.11 (0.29)	—
Task9 min2	1.21 (0.20)	0.0067

### 3.2 Phase II: Paired Analysis Between Viewing, Tasks, and 24-hour

During Phase II, participants had an average DFA *α*1 = 1.09 (SD = 0.14). Scatterplots of the epochs showed a flat trend in 82% (18 out of 22) of our participants; 18% (4 out of 22) showed a dip for 200 of the 2-minute intervals (approximately 6.67 h). The scatterplots are available in the Supplementary Material. 5 out of 9 comparisons showed significances between participants’ 24-hour DFA *α*1 and that of tasks. DFA *α*1 during the task segments ranged from 1.13 (SD = 0.25) to 1.27 (SD = 0.16) (Table 6). In contrast, 0 out of 9 comparisons showed significances between participants’ 24-hour DFA *α*1 and that of viewing. DFA *α*1 for our 22 participants during viewing segments ranged from 0.99 (SD = 0.24) to 1.13 (SD = 0.23) (Table 6). It is worth noting that within this subset, 5 out of 17 pairs showed significant differences when switching from viewing to tasks (Supplementary Table S5). The significance level was corrected for multiple comparisons (Bonferroni correction, *p*-value threshold <0.0028). Moreover, The Phase II data on a 2-minute resolution did not present statistically significant alternating findings, however, because of the volume of data (about 600 comparisons per person) it is not feasible to present. For each participant in the study, the Supplementary Figures S1–S15 show the complete Phase II data with a 2-minute resolution to illustrate this finding.

**TABLE 6:** DFA *α*1 comparisons between 24-hour and (viewing or task). *p*-values are reported in comparison with 24-hour DFA *α*1.

	DFA α1	*p*
Viewing		
Viewing 1	0.99 (0.24)	0.091
Viewing 2	1.01 (0.23)	0.130
Viewing 3	1.02 (0.26)	0.380
Viewing 4	1.03 (0.22)	0.205
Viewing 5	1.05 (0.29)	0.731
Viewing 6	1.06 (0.28)	0.444
Viewing 7	1.12 (0.20)	0.416
Viewing 8	1.12 (0.28)	0.561
Viewing 9	1.13 (0.23)	0.300
Task		
Task 1	1.13 (0.25)	0.424
Task 2	1.25 (0.19)	<0.0028
Task 3	1.20 (0.16)	0.0059
Task 4	1.23 (0.18)	<0.0028
Task 5	1.22 (0.17)	0.0030
Task 6	1.27 (0.16)	<0.0028
Task 7	1.22 (0.22)	0.0031
Task 8	1.25 (0.15)	<0.0028
Task 9	1.26 (0.17)	<0.0028

## 4 Discussion

In our study with a population of healthy young adults, our results show that participants were responsive to unique external environments, such as viewing and tasking, and their physiological responses were captured *via* the DFA *α*1 parameter. Moreover, their dynamic responsiveness persisted over the course of our study during Phase I, even as participants likely habituated to the study environment. Our Phase I results were also compared to Phase II, where subjects did not exhibit these types of shifts on the same 2-minute resolution over the course of a 24-hour period. This suggests that the Phase I settings may be a beneficial clinical tool to capture cardiac agility and dynamicity in response to two different external states.

Our results showed that DFA *α*1 as a non-linear index of HRV did not distinguish the different affective image regimes under ultra-short recordings. Although previous work showed that there can be differences in HRV measures when comparing neutral and arousing sessions (Valenza et al., 2012) or watching positive and negative videos (Barquero-Pérez et al., 2020; Ghiasi et al., 2020), our findings are consistent with other works that showed the linear HRV metric RMSSD did not distinguish between positive and negative emotions using IAPS (Schippers et al., 2018), and non-linear HRV metric DFA *α*1 did not distinguish different emotionally arousing settings (Marín-Morales et al., 2021). We hypothesize that because pure regime participants knew to expect images of the same type, there may not have been a sufficient change in emotional context to create significant variation in their heart rate.

We also have found that DFA *α*1 deviated from normal, baseline values during the viewing segments of Phase I to more elevated values during tasks. Throughout the viewing segments of Phase I, participants’ DFA *α*1 remained within the range from 0.96 (SD = 0.25) to 1.08 (SD = 0.22). This result implies an almost perfect log-log correlation, and are values generally found in healthy subjects (Kuusela, 2013). However, DFA *α*1 shifted to elevated values throughout the task segments of Phase I, ranging from 1.17 (SD = 0.21) to 1.26 (SD = 0.25). The deviation of DFA *α*1 from 1 during tasks suggested a decrease in fluctuation and randomness in HRV (Goldberger, 1996). The differences in viewing and tasks reflected by DFA *α*1 may be due to more active participation during the task segments by engaging in cognitive processes such as performing a math test and a memory recall. Looking at DFA *α*1 at the one-minute resolution, during memory recall participants had values as high as 1.27 (SD = 0.20), whereas during the math test the highest DFA *α*1 = 1.18 (SD = 0.26). We thus further hypothesize that the elevation of DFA *α*1 that emerged during tasks is because the memory recall portion was evaluated by a research assistant in real time. This supports a previous study finding on the effects of social evaluation to stress (Schwabe et al., 2008). The consistency in DFA *α*1 across images segments was expected, as images within a segment were designed to be the same in arousal, valence, and relatedness throughout the 2 min. This suggests the difference in levels of engagement can be registered *via* the DFA parameter.

The goal of the 24-hour Holter monitoring was to compare changes in DFA *α*1 on the 2-minute resolution to the 2-minute scale during Phase I of this study, as well as to investigate the limit of DFA *α*1 in its ability to distinguish viewing and tasks. Interestingly, DFA *α*1 can distinguish between the two activities providing evidence to suggest that Phase I displays differences when compared to variations that occur during everyday living. The viewing segments compared to the task segments may be able to tease out two important and unique responses in the heart that can be meaningful depending on what you need to find (one in response to physiological arousal and the other in response to physiological habituation) as presented above. Taken together (Phase I and Phase II), the external environment appears to be a factor in how the heart responds. We also found that most participants (82%) had roughly uniform DFA *α*1 calculated with 2-minute resolution over the 24-hour period. This may allow for limited comparison between the 24-hour study (Phase II) and the 2-minute period (Phase I) that needs to be validated in future studies. Overall, our results suggested that there were notable differences in heart rate metrics during Phase I and Phase II of this study. The 24-hour Holter recording serves as a baseline for participants, and the tasks segments seem to allow for participants to exhibit a change in variability, potentially reflective of the impact of the external environment on HRV.

The findings above may be applicable in understanding clinical pathologies. Specifically, if the healthy heart can switch in response to external environments, the loss of the ability to achieve the DFA parameter switch could suggest an abnormality. The benefit of studying healthy participants is to understand how the healthy heart responds. We hypothesize that a future study might find a change in the switching ability of unhealthy participants under these same conditions. Specifically, because studies of congestive heart failure (CHF) patients have shown changes in DFA values (Zhu et al., 2014; de Morais, 2015), the magnitude of alternating behavior in the DFA exponent may be different in participants with underlying heart disease. Such observations can then possibly be used in primary care clinics where patients view images and perform simple, non-physical tasks for as few as 20 min to determine their overall HRV. The data collected this way has the advantage of being both controlled and only requiring a short time commitment for patients.

In addition to the clinical application, our findings also offer insights into the newly emerged theory of network physiology—how one’s different physiological systems coordinate and integrate to maintain health (Balague et al., 2020). As the name implies, organ systems and interactions among these are represented by nodes and edges. According to the theory, network reorganization can accompany perturbations which are then reflected in physiological parameters. Goldberger et al. showed that in patients with heart disease, their physiological abilities for fractal organization deteriorates (Goldberger et al., 2002). Our participants, who have no known heart disease, demonstrated their reorganization ability with shifts in DFA *α*1 when experiencing external perturbations. Most notably shown in Figure 3 and Figure 4, participants’ DFA *α*1 returned to baseline after every elevated task segment for a total of nine times. The origin of the elevated DFA value is elucidated on the minute-by-minute scale. During the memory recall tasks, participants reached the highest DFA values ranging from 1.21 (SD = 0.20) to 1.27 (SD = 0.20). Past studies have shown that highly arousing emotional stimuli engage sympathetic-adrenomedullary output which triggers a rapid increased in cardiovascular and noradrenergic responses (Schwabe et al., 2012). Future studies should examine to what extent changes in regime may influence autonomic balance as it relates to downstream cardiac effects.

Some nonlinear properties of cardiac interbeat intervals multifractal properties of the heart have been shown to be altered by drugs, such as beta-blockers and atropine suggesting that chemical interventions may be able to change the heart’s fractal nature (Nunes Amaral et al., 2001). Yet others recently have found low effects of beta-blockers on scaling coefficients (Castiglioni et al., 2011). In a study by Lin et al., they found the use of beta-blockers had a significant effect on patients’ DFA *α*1 (Lin et al., 2001). Our findings during Phase I when comparing DFA *α*1 viewing and task between subjects are similar in nature to Nunes Amaral et al. and Lin et al.’s findings. Specifically, the significant change occurs when switching from viewing to tasking. What this implies is that viewing images and completing tasks can provide an alternative, meaningful, and noninvasive way to probe the heart function. Our results suggest that DFA may provide complementary information to a clinical toolbox to probe cardiac function.

## 5 Limitation

One limitation of our study is the demographics of the participants. 70% (49 out of 70) of our participants were female. The imbalance of gender could impede the generalizability of this study as females could have greater parasympathetic autonomic functions than males (Abhishekh, 2013; Koenig and Thayer, 2016). Another limitation is that sleeping schedules were not adjusted in the 24-hour Holter monitoring (although only the first 20 h of data was analyzed). Balague et al. showed that circadian rhythms can influence temporal characteristics (Balague et al., 2020). Also, it is important to note that in Phase I we used Lead III for our data acquisition, and in Phase II we used Lead I for our data acquisition. These leads were chosen based on the best signal quality for the corresponding Phase of the study. Recent studies show that using different leads may impact our results (Jeyhani, 2019).

## 6 Conclusion

In conclusion, we found significant differences in the viewing versus tasks segments of this study. These differences are magnified on the one-minute temporal resolution scale and persist when compared to 24-hour analyses. We believe that the emergence of the alternating DFA *α*1 behavior in response to a varied, external environment could provide important insight into cardiovascular health and fitness in a noninvasive and practical way.

## Data Availability

The raw data supporting the conclusion of this article will be made available by the authors, without undue reservation.

## Supplementary Material

Supplementary Table S1 DFA α1 comparisons during each image viewing type within pure regime participants.

Supplementary Table S2 DFA α1 two-minute comparison within tasks and within viewing. *p*-values are reported in comparison with previous activity. All numbers except *p*-values are presented as mean (SD).

Supplementary Table S3 DFA α1 one-minute comparison during Phase I. *p*-values are reported in comparison with previous activity. All numbers except *p*-values are presented as mean (SD).

Supplementary Table S4 DFA α1 one-minute comparison within viewing. All numbers except *p*-values are presented as mean (SD).

Supplementary Table S5 DFA α1 two-minute comparison in Phase II participants. *p*-values are reported in comparison with previous activity. All numbers except *p*-values are presented as mean (SD).
