# Supplementary material for: Heart Rate Variability in Healthy Subjects During Monitored, Short-Term Stress Followed by 24-hour Cardiac Monitoring
Source: Front Physiol. 2022 Jun 13;13:897284. doi: 10.3389/fphys.2022.897284 (PMC9234740; doi:10.3389/fphys.2022.897284)
Supplement: Supplementary file 5 [file Table1.docx]

**Supplemental Table 1.** DFA α1 comparisons during each image viewing type within pure regime participants.

|  | ***p*** |
| --- | --- |
| **Negative1 - Categorical1** | 0.405 |
| **Negative1- Neutral1** | 0.360 |
| **Categorical1 - Neutral1** | 0.367 |
|  |  |
| **Negative2 – Categorical2** | 0.835 |
| **Negative2- Neutral2** | 0.320 |
| **Categorical2- Neutral2** | 0.462 |
|  |  |
| **Negative3 – Categorical3** | 0.771 |
| **Negative3- Neutral3** | 0.679 |
| **Categorical3- Neutral3** | 0.931 |

**Supplemental Table 2.** DFA α1 two-minute comparison within tasks and within viewing. p-values are reported in comparison with previous activity. All numbers except p-values are presented as mean (SD).

|  | DFA α1 | *p* |
| --- | --- | --- |
| Viewing | | |
| Viewing 1 | 0.96 (0.25) | - |
| Viewing 2 | 0.99 (0.25) | 0.151 |
| Viewing 3 | 1.01 (0.26) | 0.581 |
| Viewing 4 | 1.02 (0.22) | 0.495 |
| Viewing 5 | 1.05 (0.26) | 0.160 |
| Viewing 6 | 1.04 (0.27) | 0.249 |
| Viewing 7 | 1.07 (0.23) | 0.416 |
| Viewing 8 | 1.07 (0.27) | 0.631 |
| Viewing 9 | 1.08 (0.22) | 0.532 |
| Task | | |
| Task 1 | 1.17 (0.21) | - |
| Task 2 | 1.23 (0.21) | 0.171 |
| Task 3 | 1.19 (0.19) | 0.057 |
| Task 4 | 1.22 (0.20) | 0.062 |
| Task 5 | 1.20 (0.20) | 0.133 |
| Task 6 | 1.26 (0.19) | 0.036 |
| Task 7 | 1.26 (0.25) | 0.799 |
| Task 8 | 1.19 (0.19) | 0.279 |
| Task 9 | 1.18 (0.18) | 0.812 |

**Supplemental Table 3.** DFA α1 one-minute comparison during Phase I. *p*-values are reported in comparison with previous activity. All numbers except *p*-values are presented as mean (SD).

|  | DFA α1 | *p* |
| --- | --- | --- |
| Viewing1 min1 | 0.99 (0.29) | - |
| Viewing1 min2 | 0.91 (0.27) | 0.008 |
| Task1 min1 | 1.01 (0.24) | 0.034 |
| Task1 min2 | 1.25 (0.22) | <0.001 |
| Viewing2 min1 | 1.00 (0.26) | <0.001 |
| Viewing2 min2 | 0.97 (0.26) | 0.285 |
| Task2 min1 | 1.11 (0.27) | <0.001 |
| Task2 min2 | 1.25 (0.20) | <0.001 |
| Viewing3 min1 | 1.04 (0.29) | <0.001 |
| Viewing3 min2 | 0.97 (0.30) | 0.053 |
| Task3 min1 | 1.07 (0.28) | 0.002 |
| Task3 min2 | 1.22 (0.20) | <0.001 |
| Viewing4 min1 | 1.02 (0.26) | <0.001 |
| Viewing4 min2 | 1.01 (0.27) | 0.639 |
| Task4 min1 | 1.15 (0.32) | <0.001 |
| Task4 min2 | 1.22 (0.22) | 0.085 |
| Viewing5 min1 | 1.06 (0.29) | <0.001 |
| Viewing5 min2 | 1.02 (0.28) | 0.337 |
| Task5 min1 | 1.08 (0.24) | 0.100 |
| Task5 min2 | 1.24 (0.22) | <0.001 |
| Viewing6 min1 | 1.05 (0.32) | <0.001 |
| Viewing6 min2 | 1.00 (0.27) | 0.174 |
| Task6 min1 | 1.18 (0.26) | <0.001 |
| Task6 min2 | 1.27 (0.20) | 0.004 |
| Viewing7 min1 | 1.09 (0.28) | <0.001 |
| Viewing7 min2 | 1.02 (0.28) | 0.058 |
| Task7 min1 | 1.14 (0.28) | 0.003 |
| Task7 min2 | 1.26 (0.23) | <0.001 |
| Viewing8 min1 | 1.09 (0.28) | <0.001 |
| Viewing8 min2 | 1.03 (0.28) | 0.048 |
| Task8 min1 | 1.12 (0.29) | 0.020 |
| Task8 min2 | 1.22 (0.19) | 0.002 |
| Viewing9 min1 | 1.11 (0.25) | 0.002 |
| Viewing9 min2 | 1.04 (0.28) | 0.035 |
| Task9 min1 | 1.11 (0.29) | 0.007 |
| Task9 min2 | 1.21 (0.20) | 0.007 |

**Supplemental Table 4**. DFA α1 one-minute comparison within viewing. All numbers except *p*-values are presented as mean (SD).

|  | DFA α1 | *p* |
| --- | --- | --- |
| Viewing1 min1 | 0.99 (0.29) | - |
| Viewing1 min2 | 0.91 (0.27) | 0.008 |
| Viewing2 min1 | 1.00 (0.26) | - |
| Viewing2 min2 | 0.97 (0.26) | 0.285 |
| Viewing3 min1 | 1.04 (0.29) | - |
| Viewing3 min2 | 0.97 (0.3) | 0.053 |
| Viewing4 min1 | 1.02 (0.26) | - |
| Viewing4 min2 | 1.01 (0.27) | 0.639 |
| Viewing5 min1 | 1.06 (0.29) | - |
| Viewing5 min2 | 1.02 (0.28) | 0.337 |
| Viewing6 min1 | 1.05 (0.32) | - |
| Viewing6 min2 | 1.00 (0.27) | 0.174 |
| Viewing7 min1 | 1.09 (0.28) | - |
| Viewing7 min2 | 1.02 (0.28) | 0.058 |
| Viewing8 min1 | 1.09 (0.28) | - |
| Viewing8 min2 | 1.03 (0.28) | 0.048 |
| Viewing9 min1 | 1.11 (0.25) | - |
| Viewing9 min2 | 1.04 (0.28) | 0.035 |

**Supplemental Table 5.** DFA α1 two-minute comparison in Phase II participants. *p*-values are reported in comparison with previous activity. All numbers except *p*-values are presented as mean (SD).

|  | DFA α1 | *p* |
| --- | --- | --- |
| Viewing 1 | 0.99 (0.24) | - |
| Task 1 | 1.13 (0.25) | 0.26 |
| Viewing 2 | 1.01 (0.23) | 0.23 |
| Task 2 | 1.25 (0.19) | <0.0028 |
| Viewing 3 | 1.02 (0.26) | 0.02 |
| Task 3 | 1.20 (0.16) | 0.03 |
| Viewing 4 | 1.03 (0.22) | <0.0028 |
| Task 4 | 1.23 (0.18) | <0.0028 |
| Viewing 5 | 1.05 (0.29) | <0.0028 |
| Task 5 | 1.22 (0.17) | 0.03 |
| Viewing 6 | 1.06 (0.28) | 0.02 |
| Task 6 | 1.27 (0.16) | 0.01 |
| Viewing 7 | 1.12 (0.2) | 0.01 |
| Task 7 | 1.22 (0.22) | 0.04 |
| Viewing 8 | 1.12 (0.28) | 0.08 |
| Task 8 | 1.25 (0.15) | 0.06 |
| Viewing 9 | 1.13 (0.23) | 0.10 |
| Task 9 | 1.26 (0.17) | <0.0028 |
